# Diagnosing FSGS without kidney biopsy – a novel *INF2*-mutation in a family with ESRD of unknown origin

**DOI:** 10.1186/s12881-016-0336-9

**Published:** 2016-10-12

**Authors:** Johannes Münch, Maik Grohmann, Tom H. Lindner, Carsten Bergmann, Jan Halbritter

**Affiliations:** 1 Department of Internal Medicine, Division of Nephrology, University Clinic Leipzig, Liebigstrasse 20, 04103 Leipzig, Germany; 2 Center for Human Genetics, Bioscientia, Konrad-Adenauer-Straße 17, 55218 Ingelheim, Germany

**Keywords:** Focal segmental glomerulosclerosis, FSGS, Nephrotic syndrome, *INF2*

## Abstract

**Background:**

Patients on renal replacement therapy are often unaware of their underlying condition and hence suffer from so-called end-stage renal disease (ESRD) of unknown origin. However, an exact diagnosis is not only important for better estimating the prognosis, but also when preparing for kidney transplantation. Whilst patients with FSGS without a confirmed genetic cause have a high recurrence rate in the transplanted organ, patients with a mutation generally exhibit no recurrence and have a good prognosis. Furthermore, renal biopsy, which may be helpful for differential diagnosis, is usually contraindicated in end-stage kidneys. We here present the case of familial ESRD of unknown origin, which could be resolved by targeted genetic testing prior to planning of kidney transplantation.

**Case presentation:**

A 32-year-old female with ESRD and nephrotic range proteinuria was admitted to our clinic. Family-history revealed that both mother and maternal grandmother had ESRD of unknown origin. As renal biopsy was impossible due to atrophic kidneys, we performed mutation analysis of genes known for dominant forms of FSGS and found a novel heterozygous mutation of *INF2* (c.485 T > C, p.Leu162Pro). The same mutation could be detected in the index patient’s mother (ESRD at age 50) and three brothers with normal serum-creatinine but mid or low range proteinuria.

**Conclusions:**

Genetic testing is warranted in families with ESRD of unknown origin and may provide a robust diagnosis even without kidney biopsy. It will help detecting relatives at risk who have to be excluded from potential kidney donation and who may benefit from timely initiation of protective measures in order to slow down disease progression.

## Background

Unfortunately, ignorance of the underlying renal condition is a common phenomenon among patients on renal replacement therapy. This is often due to late clinical presentation at stages of advanced disease, when important diagnostic tools, such as kidney biopsy, are no longer possible for diminished organ size and consecutive risk of bleeding. Focal segmental glomerulosclerosis (FSGS) is such an underlying, potentially underdiagnosed, condition. FSGS is derived from its histological appearance upon kidney biopsy and represents a strictly descriptive term that comprises heterogeneous disorders which altogether represent a major cause of nephrotic syndrome, progressive renal failure, and end-stage renal disease (ESRD) [1]. It is characterized by focally occurring glomeruli with segmental capillary sclerosis and podocytic foot-process effacement upon light and electron microscopy. Apart from secondary causes, such as obesity, infections, drugs, and others, more than 30 single-gene causes have been identified over the past decades [2]. By unraveling those genetic causes, the knowledge on podocytes and the essential role of the actin cytoskeleton for the integrity of the glomerular filtration barrier has expanded dramatically [3]. The most common cause of autosomal-dominant FSGS in adults is thought to be due to mutations in the gene *INF2* [4], which encodes a member of the so called formin family of proteins that are supposed to sever actin filaments and accelerate their polymerization and depolymerization [5]. *INF2*-gene mutations were identified in both FSGS and Charcot-Marie-Tooth disease, a hereditary motor and sensory neuropathy that is characterized by peripheral nerval demyelination [6]. Virtually all disease-causing mutations of the *INF2* gene associated with FSGS have been found within exons coding for its highly conserved diaphanous-inhibitory domain (DID), which serves as a regulator for polymerization and depolymerization of actin filaments [7]. In contrast to many other genetic forms of FSGS, patients with *INF2*-mutations do usually not manifest before adolescence or early adulthood and age at ESRD can vary significantly (published data range 12–70 years) [6–8].

## Case presentation

**Fig. 1:**
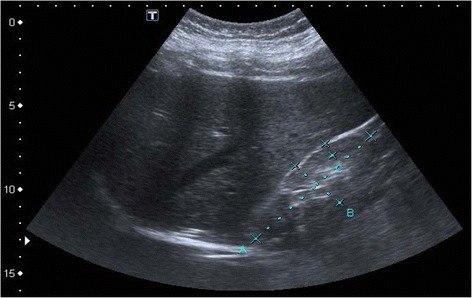
Abdominal ultrasonography of index patient. Right kidney is overall diminished in size (a-b: 88 × 33 mm, c: renal parenchyma 8 mm) and appears hyperechogenic compared to liver parenchym

The index patient, a 32-year-old Caucasian female with proteinuria (5.7 g/g creatinine) and impaired renal function (serum creatinine 222 μmol/l, eGFR-CKD-EPI 24 ml/min/1.73 m^2^), was admitted to our nephrology department. Except for edema of the legs, physical examination showed no further abnormalities, in particular, no indication of a sensorimotor or other neurological disorder. Both kidneys presented atrophic on ultrasonography (Fig. 1) with a hyperechogenic and diminished renal parenchyma. We therefore decided not to perform kidney biopsy for risk of hemorrhagic complications. Blood pressure was slightly elevated, in the range of 140–150 mmHg systolic. No other mentionable laboratory abnormalities, including normal auto-antibody screening (anti-nuclear = ANA, anti-neutrophil cytosplasmatic = ANCA), were noted.

**Fig. 2:**
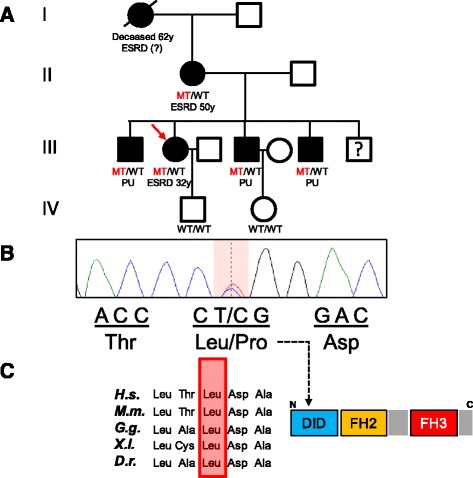
**a** Pedigree with affected family members denoted by black fillings, the index patient (III-2) is indicated by a red arrow. **b** Heterozygous *INF2*-mutation (c.485 T > C) present in all affected family members, indicated as MT for mutant and WT for wildtype. **c** Highly conserved amino acid-residue leucine (p.162), altered to proline in affected family members, located within the N–terminal DID-domain. Legend: ESRD, end-stage renal disease; *H.s., Homo sapiens; M.m., Mus musculus; G.g., Gallus gallus; X.l., Xenopus laevis; D.r., Danio reri*o; PU, proteinuria

Family history yielded ESRD of unknown etiology in her 53-year-old mother, since the age of 50, and a non-specified kidney disease in her already deceased maternal grandmother. In suspicion of a dominantly inherited disorder with progressive nephrotic syndrome, we initiated genetic testing for the most common single-gene causes of dominant familial FSGS. Sequencing of actinin alpha 4 *(ACTN4*) was unremarkable. Direct sequencing of inverted formin 2 (*INF2*), however, showed a heterozygous nucleotide exchange from thymine to cytosine (c.485 T > C) in exon 3, resulting in the substitution of a highly conserved leucine to proline at amino acid position 162 (p.Leu162Pro) (Fig. 2). Upon confirmatory analysis, we could prove familial segregation and the same mutation was present in her 53-year-old mother. We subsequently screened all available family members for the presence of proteinuria and identified increased levels of urinary albumin in three brothers of the index patient, none of them with remarkable rise of serum creatinine (III-1 33 years: 1600 mg/g creatinine, III-3 28 years: 451 mg/g creatinine, III-4 20 years: 177 mg/g creatinine). The familial *INF2*-mutation was detected in all three affected brothers (Fig. 2, Table 1), who were consecutively started on treatment with an ACE-inhibitor and informed about general measures of kidney protection. In light of the genetic diagnosis, the affected brothers were excluded as potential living kidney donors for the index patient and her mother. Unfortunately, the index patient rapidly progressed to ESRD with an eGFR of 15 ml/min within 10 months of first presentation.

**Table 1:** Clinical characteristics of affected family members

Individual/age	Genetic diagnosis^a^	Age at ESRD	Proteinuria (mg/g Crea)	S-creatinine (μmoL)/eGFR (ml/min/1.73 m^2^)	Renal biopsy/sonography
Index (III-2) 32 years	c.485 T > C p.Leu162Pro (het)	32	5700	222/15	NA/atrophic kidneys
Mother (II-1) 53 years	c.485 T > C p.Leu162Pro (het)	50	NA	>300/<15	NA/atrophic kidneys
Bother1 (III-1) 33 years	c.485 T > C p.Leu162Pro (het)	NA	1600	95/98	NA/normal kidneys
Brother2 (III-3) 28 years	c.485 T > C p.Leu162Pro (het)	NA	451	78/116	NA/normal kidneys
Brother3 (III-4) 20 year	c.485 T > C p.Leu162Pro (het)	NA	177	77/124	NA/normal kidneys

*ESRD* end-stage renal disease, *het* heterozygous, *NA* not annotated, *yr* years

^a^cDNA mutations are numbered according to human cDNA reference sequence NM_022489.3 (*INF2*), where +1 corresponds to the A of ATG start translation codon

## Conclusions

We here report a novel *INF2*-mutation (c.485 T > C, p.Leu162Pro) in a family with ESRD of previously unknown etiology. As in virtually all patients with FSGS due to mutated *INF2*, the detected mutation is located within the first exons and results in an amino acid change within the functionally important N-terminal DID [9]. Involvement of the same codon was previously described in a study by Caridi et al. (2014), however, resulting in yet another aa-substitution (p.Leu162Arg) [7]. As kidney biopsy was rejected for risks of bleeding, this family illustrates nicely the diagnosis of FSGS, solely based on a robust molecular genetic diagnosis. Based on the initial findings of the index patient and her mother, we successfully screened for further family members at risk and found three brothers with normal kidney function but asymptomatic proteinuria (<2 g/g creatinine). In all five affected family members alive, the familial *INF2*-mutation was found in heterozygous state (Fig. 2), while family members without proteinuria were tested wildtype. Interestingly, the clinical course was markedly variable, with the most severe affection in the index patient (ESRD at 32). At this point, it remains speculative whether unidentified genetic or environmental modifiers may account for these phenotypic differences.

As previously shown by Sun et al. (2013), the resulting dysfunction of INF2 is responsible for a deranged structure of the cytoskeleton, leading to an abnormal distribution of podocin and nephrin as important components of the podocytic slit membrane [10]. Disturbed intra- and transcellular transportation of proteins due to an impaired polymerization and depolymerization of actin filaments may be the reason for these histological findings [10]. To date, there is no causative medical treatment of FSGS due to defective INF2. Renal transplantation, however, can be considered a curative treatment for patients without neurological manifestation (Charcot-Marie-Tooth disease), provided the donor kidney expresses functional INF2. Therefore, a thorough evaluation of potential living kidney donors is indispensable. As illustrated in our family, the clinical picture can be extremely variable. A definite and valid exclusion of the disease will only be possible by genetic testing. In case of timely diagnosis at an early stage of disease (III-1, III-3, III-4), anti-proteinuric medication with inhibitors of the renin-angiotensin-aldosterone-system should be initiated. Apart from ACE-inhibitors and AT1-blockers, aldosterone antagonists (e.g., spironolactone) might offer an alternative therapeutic option, as aldosterone seems to also have a direct influence on several podocytic processes, like the generation of stress fibers and inducing the disassembly of cortical actin- and cell-cell-junctions [11, 12].

In conclusion, we strongly suggest genetic testing in younger patients with a positive family history but ESRD of unknown origin: i) targeted genetic testing based on clinical suspicion provides a reasonable likelihood of detecting the underlying condition even when kidney biopsy is contraindicated, ii) knowledge of the causative renal disorder is highly informative for risk-assessment and planning of kidney transplantation (e.g., risk of recurrence), iii) consecutive detection of family members at risk will help to exclude potential living kidney donors within the family, iv) timely initiation of reno-protective measures will help affected family members to slow down disease progression and protract or even avoid ESRD.

## Abbreviations

A: Adenine
ACE-inhibitor: Inhibitor of angiotensin converting enzyme
*ACTN4*: Actinin alpha 4(the gene)
Ala: Alanine
ANA: Antinuclear antibody
ANCA: Anti-neutrophil cytoplasmic antibody
Arg: Arginine
Asp: Aspartic acid
AT1-blocker: Angiotensin typ 1 receptor blocker
C: Cytosine
cDNA: Complementary deoxyribonucleic acid
CKD-EPI: Chronic kidney disease epidemiology collaboration
Crea: Creatinine
Cys: Cysteine
D.r.: Danio rerio (lat.)
DID: Diaphanous inhibitory domain
e.g.,: Exempli gratia (lat.)/for example
eGFR: Estimated glomerular filtration rate
ESRD: End-stage renal disease
FH2: Formin homology 2
FH3: Formin homology 3
FSGS: Focal segmental glomerulosclerosis
G: Guanine
G.g.: Gallus gallus (lat.)
H.s.: Homo sapiens (lat.)
het.: Heterozygous
*INF2*: Inverted formin 2 (the gene)
Leu: Leucine
M.m.: Mus musculus (lat.)
MT: Mutant
NA: not annotated
Pro: Proline
PU: Proteinuria
T: Thymine
Thr: Threonine
WT: Wildtype
yr: Years
